# Photonic neural probe enabled microendoscopes for light-sheet light-field computational fluorescence brain imaging

**DOI:** 10.1117/1.NPh.11.S1.S11503

**Published:** 2024-02-06

**Authors:** Peisheng Ding, Hannes Wahn, Fu-Der Chen, Jianfeng Li, Xin Mu, Andrei Stalmashonak, Xianshu Luo, Guo-Qiang Lo, Joyce K. S. Poon, Wesley D. Sacher

**Affiliations:** a Max Planck Institute of Microstructure Physics, Halle, Germany; b University of Toronto, Department of Electrical and Computer Engineering, Toronto, Ontario, Canada; c Max Planck-University of Toronto Centre for Neural Science and Technology, Toronto, Ontario, Canada; d Advanced Micro Foundry Pte. Ltd., Singapore

**Keywords:** microendoscopes, neurophotonics, integrated optics, light-sheet fluorescence microscopy, lensless imaging, neural probes

## Abstract

**Significance:**

Light-sheet fluorescence microscopy is widely used for high-speed, high-contrast, volumetric imaging. Application of this technique to *in vivo* brain imaging in non-transparent organisms has been limited by the geometric constraints of conventional light-sheet microscopes, which require orthogonal fluorescence excitation and collection objectives. We have recently demonstrated implantable photonic neural probes that emit addressable light sheets at depth in brain tissue, miniaturizing the excitation optics. Here, we propose a microendoscope consisting of a light-sheet neural probe packaged together with miniaturized fluorescence collection optics based on an image fiber bundle for lensless, light-field, computational fluorescence imaging.

**Aim:**

Foundry-fabricated, silicon-based, light-sheet neural probes can be packaged together with commercially available image fiber bundles to form microendoscopes for light-sheet light-field fluorescence imaging at depth in brain tissue.

**Approach:**

Prototype microendoscopes were developed using light-sheet neural probes with five addressable sheets and image fiber bundles. Fluorescence imaging with the microendoscopes was tested with fluorescent beads suspended in agarose and fixed mouse brain tissue.

**Results:**

Volumetric light-sheet light-field fluorescence imaging was demonstrated using the microendoscopes. Increased imaging depth and enhanced reconstruction accuracy were observed relative to epi-illumination light-field imaging using only a fiber bundle.

**Conclusions:**

Our work offers a solution toward volumetric fluorescence imaging of brain tissue with a compact size and high contrast. The proof-of-concept demonstrations herein illustrate the operating principles and methods of the imaging approach, providing a foundation for future investigations of photonic neural probe enabled microendoscopes for deep-brain fluorescence imaging *in vivo*.

## 1 Introduction

The goal of a comprehensive understanding of neural circuitry and the neuronal activities underlying behavior has driven the development of imaging technologies to observe increasingly large tissue volumes with high spatiotemporal resolution.^1^^–^^5^ Further developments are needed to fully utilize the ever-increasing sophistication of optogenetic tools, which include multi-color opsins for optically evoking and silencing neuronal activity^6^^,^^7^ and genetically encoded fluorescent indicators of activity (via calcium or voltage imaging),^8^^–^^11^ all with cell-type specificity. Among the plethora of single-photon and multiphoton fluorescence imaging techniques, light-sheet fluorescence microscopy (LSFM) has become an established technique for high-speed, high-resolution, volumetric optical imaging of live specimens.^12^^–^^15^ Standard LSFM involves sample illumination with a scanned light sheet, which exclusively excites a thin slice of the fluorescent sample, with the slice aligned to the focal plane of fluorescence collection optics, reducing the background fluorescence, photobleaching, and photoxicity relative to widefield imaging.^16^^,^^17^ However, conventional LSFM requires two objectives placed orthogonally for light sheet delivery and fluorescence collection, imposing geometric constraints that limit the compatibility with fluorescence brain imaging of non-transparent, awake, and behaving animals. Demonstrations of light-sheet brain imaging of non-transparent organisms have been limited to superficial depths in mouse brain tissues *in vitro*^18^ and *in vivo*,^19^ in addition to a demonstration of a millimeter-scale LSFM endoscope assembly with a millimeter-scale prism and two gradient-index (GRIN) lenses.^20^ Overall, a minimally invasive LSFM approach compatible with both deep-brain imaging and awake, behaving animals has remained elusive, and the benefits of achieving this goal, especially in concert with functional fluorescence imaging, offer an opportunity for sizeable gains in spatiotemporal resolution and/or imaging volume relative to current deep-brain imaging approaches.

**Fig. 1:**
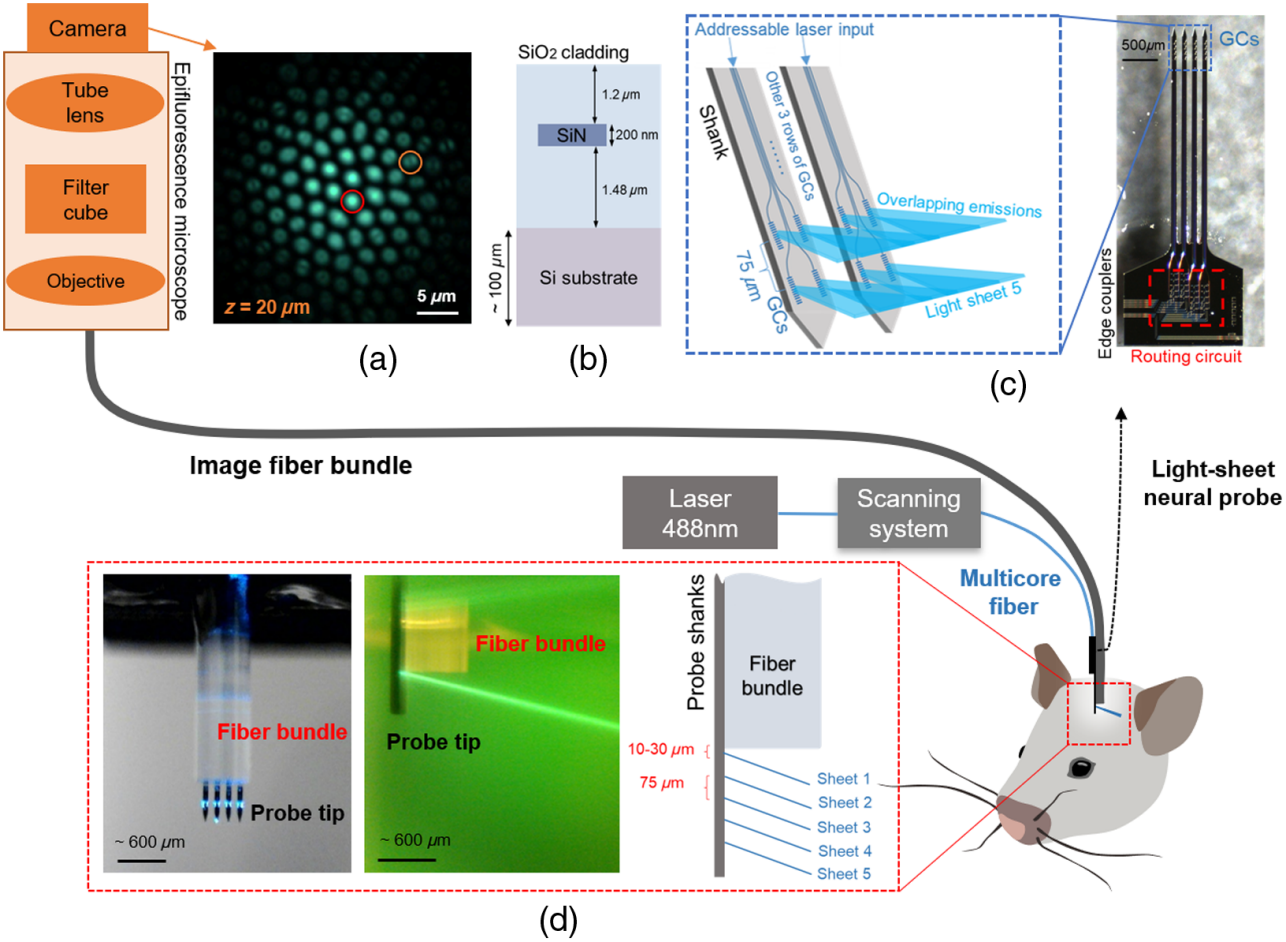
Light-sheet light-field microendoscope system. The imaging system consists of a microendoscope formed from an image fiber bundle (for fluorescence collection) with an attached implantable photonic neural probe at the distal fiber end (for delivery of excitation light) and a widefield/epifluorescence microscope for imaging fluorescence patterns at the proximal fiber bundle facet. Laser light (λ=488  nm) is coupled to the on-chip silicon nitride (SiN) waveguide circuitry of the neural probe via a custom visible-light multicore optical fiber with individual cores addressed by an optical scanning system; the probe subsequently emits addressable light sheets at the tip of the microendoscope. The mouse illustration was adapted from Ref. 22. (a) Proximal fiber bundle facet raw image of a fluorescent bead (10  μm diameter) at an axial distance (z) of 20  μm from the distal fiber bundle facet showing the defocused fluorescence collection and increased coupling to higher-order optical modes in the peripheral fiber bundle cores within the defocused bead image. The fiber bundle image contains both intensity and angular information of the incident light, enabling volumetric reconstructions via light-field imaging algorithms.^23^ Two fiber bundle cores showing different optical mode patterns are encircled. (b) Cross-section illustration of the neural probe chips (not to scale). (c) Micrograph of a fabricated neural probe chip (right) and illustration of light sheet generation from the SiN grating coupler (GC) emitters (left). Five rows of GCs were integrated onto 100-μm thick, 3-mm-long shanks, and light sheets are synthesized from the overlapping emissions from the GCs within each row. To achieve wide, thin sheets, the GCs were designed to emit beams of large divergence along the sheet width axis and small divergence along the sheet thickness axis. (d) Photographs of the microendoscope emitting a light sheet: front view in air (left) and side view in fluorescein (middle). Schematic showing the relative positions of the light sheets and fiber bundle in the microendoscope (right). The micrographs in panels (a) and (c), fluorescein image in panel (d), and neural probe illustrations in panels (c) and (d) were adapted from our conference abstract; see Ref. 24.

To surmount the geometric constraints of conventional LSFM, we recently reported implantable photonic neural probes that synthesize light sheets in brain tissue from arrays of nanophotonic waveguides and grating couplers (GCs), thereby miniaturizing the illumination optics.^21^ The prototype probes, with a thickness 50 to 92  μm, incorporated rows of silicon nitride (SiN) GCs for synthesizing 5 to 10 addressable light sheets [Fig. 1(c)]. In a series of imaging experiments, neural-probe enabled LSFM was demonstrated with large contrast enhancements compared with conventional widefield epifluorescence microscopy in fixed, *in vitro*, and *in vivo* mouse brain tissues. Although the illumination optics were effectively miniaturized, the collection optics in the system relied on a bulky widefield microscope, limiting applicability to deep-brain fluorescence imaging experiments. For deep-brain imaging, the collection optics should be implanted to enable imaging at depths beyond the optical attenuation length in brain tissue. GRIN lenses are often used in implantable endoscopes, but their large diameter (typically >1  mm) results in significant tissue displacement during implantation.^25^^,^^26^ Alternatively, fiber-optic microendoscopy has emerged as a sophisticated implantable technique that uses a narrow optical fiber image bundle (often ≈300 to 650  μm in diameter) to transmit images collected by the distal end of the fiber to its proximal end, enabling imaging access to deep regions of the body.^27^ The utility of image fiber bundles has been widely demonstrated in living tissue experiments and implemented in clinically relevant settings, such as bronchoscopy and ovarian cancer detection.^28^^,^^29^ Despite its advantages, fiber bundle imaging involves coaxial light delivery and collection through the bundle (epi-illumination) and, similar to widefield microscopes, suffers from low image contrast in addition to relatively high photobleaching and phototoxicity when used for fluorescence imaging.

In this work, we propose and demonstrate an implantable fluorescence imaging microendoscope system with a photonic neural probe for light-sheet illumination attached to an optical fiber bundle for fluorescence collection. We reported our initial results in Ding et al., 2023.^24^ Our imaging system combines light-sheet neural probes with the fiber-bundle light-field moment imaging technique,^23^^,^^30^^,^^31^ allowing volumetric image reconstructions from each captured image frame and eliminating the need for a tunable focus element at the distal fiber end. The developed light-sheet light-field (LSLF) microendoscopic imaging system, as shown in Fig. 1, separates the bulk imaging optics and image sensor from the sample and is compatible with high-performance cameras, such as back-illuminated, cooled, scientific CMOS (sCMOS) cameras. The diameter of the image fiber bundle (650  μm) is considerably smaller than a typical implantable GRIN lens,^32^^,^^33^ and the shanks of the neural probe (each approximately 65  μm wide and 100  μm thick) account for only around 8% of the overall implanted volume. By combining the advantages of fiber-bundle light-field imaging with light-sheet neural probes, the microendoscopes demonstrated herein open an avenue toward a new class of minimally invasive volumetric imaging tools for deep-brain fluorescence imaging.

## 2 Results

### 2.1 Microendoscope Design and Operation

The LSLF microendoscope is illustrated in Fig. 1 and consists of an image fiber bundle (FIGH 30-650S, 650  μm diameter, 30,000 cores, Fujikura) attached to a light-sheet neural probe aligned parallel to the fiber axis. The neural probe extends beyond the distal fiber bundle facet and emits light sheets that can be addressed/selected, with each sheet selectively illuminating a planar section of the imaging volume underneath the fiber bundle. As each sheet sequentially illuminates the sample, emitted fluorescence is incident on the distal facet of the fiber bundle, forming a defocused pattern that is imaged by a conventional widefield microscope (equipped with an sCMOS camera) at the proximal end of the fiber bundle; see Fig. 1(a). Importantly, depth information of fluorescent sources is encoded in both the number of cores to which fluorescence is coupled (i.e., the spot size) and the distribution of transverse optical modes excited within the cores,^23^ each core supporting several optical modes. The diverging light from a fluorescent source within the imaging volume preferentially excites the fundamental and low-order modes of fiber bundle cores inline with the source and excites higher order modes of off-axis/laterally offset cores. In their 2019 publication, Orth et al.^23^ demonstrated a light-field moment imaging algorithm to reconstruct volumetric images from defocused fiber bundle images, and here, we apply this algorithm to reconstruct volumetric images for each light sheet emitted by the neural probe - circumventing the requirement for a tunable focus optical element to scan a focal plane through the imaging volume. The light-field reconstruction algorithm is outlined in Sec. 4.4 and Fig. S7 in the Supplementary Material.

The light-sheet neural probe design, fabrication, and operation are detailed in our previous work.^21^ Briefly, the neural probes were fabricated on 200-mm diameter silicon (Si) wafers at Advanced Micro Foundry (AMF). A series of plasma enhanced chemical vapor deposition, deep ultraviolet photolithography, reactive ion etching, and planarization steps were used to define 200-nm nominally thick SiN waveguides with ${\mathrm{SiO}}_{2}$ cladding patterned onto the Si wafers. Deep trenches were etched to define the probe shapes and facets for fiber-coupling of light onto the probe chips. Finally, backgrinding was performed to thin the wafers to ≈100  μm [Fig. 1(b)]. Each neural probe consists of four 3-mm long implantable needles (“shanks”) and a larger base region. An array of SiN waveguide edge couplers on the base of the probe couples light onto the chip from a custom visible-light multicore fiber,^34^ with each edge coupler aligned to a core of the fiber. A routing network photonic circuit consisting of splitting and crossing devices splits the light from each edge coupler into an array of waveguides that guide light down the shanks to a row of GCs, where the light is emitted. The overlapping emissions from each row of GCs synthesizes a light sheet [Fig. 1(c)], and each row of GCs is connected to a separate input edge coupler. Addressing of the various light sheets is achieved by directing laser light to different cores of the multicore fiber, and hence different edge couplers, using an external, computer-controlled, free-space optical scanning system.^35^^,^^36^

The prototype light-sheet neural probes were designed for a wavelength (λ) of 488 nm to enable excitation of commonly used fluorophores such as green fluorescent protein (GFP) and green calcium/voltage indicators.^21^ Throughout this work, the neural probes and microendoscopes were characterized and operated with an input laser of λ=488  nm. Adjustments to the dimensions of the on-chip photonic components/circuitry can shift the operating wavelength to green, red, or near-infrared wavelengths for excitation of other fluorophores. The neural probes emitted five addressable light sheets at different depths below the image fiber bundle facet. The output emission angle of the GCs, and hence the sheets, was ≈20  deg relative to the normal of the shanks, and the light-sheet illumination was oblique to the fiber bundle facet; see Fig. 1(d). The GC emission angle is dependent on the grating period and duty cycle, and we estimate that future designs with smaller periods should support angles down to ≈5  deg.

The detailed process of attaching the neural probe to the fiber bundle is elaborated in Sec. 4.2 and Note 1 in the Supplementary Material. To reduce the size of the implant, the coating of the fiber bundle was removed at the distal end. Accounting for the four neural probe shanks (each ≈65  μm in width and 100  μm in thickness) and the 650  μm fiber bundle diameter, the cross-section area of our microendoscope prototype is about $0.358\;{\mathrm{mm}}^{2}$ and >7× smaller than that of the 1.8 to 2 mm diameter implantable GRIN lenses often used in miniature head-mounted fluorescence microscopes for mice.^5^^,^^26^^,^^37^^,^^38^

Throughout this work, three LSLF microendoscopes were packaged and tested, referred to hereafter as microendoscopes 1 to 3. The microendoscopes had similar light sheet properties and optical transmissions. Microendoscope 1 was used for detailed characterization of the light sheet properties (Sec. 4.3) and imaging of fluorescent beads suspended in agarose (Sec. 2.3). Microendoscope 2 was used for imaging 3D-printed microstructures for resolution estimates (Sec. 2.2), and microendoscope 3 was used for imaging of fixed brain tissue (Sec. 2.3).

After packaging of microendoscope 1, the measured optical transmission of the light-sheet neural probe, defined here as the ratio of the emitted light sheet power from the probe and the input optical power to the scanning system, ranged from −22 to −32  dB, similar to our previously reported results.^21^ Approximately 2 dB of the optical loss was attributed to the scanning system, and we estimate that the alignment drift due to epoxy shrinkage during and after multicore fiber attachment was the primary cause of transmission variations among the sheets. In addition, the emitted light sheet profiles were characterized via fluorescence imaging, first, in a fluorescein solution, and second, using a coverslip with a fluorescent thin film positioned in close proximity to the probe. The characterization procedure is detailed in Sec. 4.3, and the results are summarized in Note 2 in the Supplementary Material. The averaged full width at half maximum (FWHM) sheet thickness in air was 11, 17, and 20  μm for light-sheet propagation distances of 100, 300, and 500  μm, respectively.

### 2.2 Microendoscope Resolution Estimates via Imaging of 3D-Printed Microstructures

As an initial evaluation of the LSLF microendoscope resolution, we imaged microstructures 3D printed using a two-photon laser writer (Photonic Professional GT2, Nanoscribe GmbH). As the structures were printed via two photon polymerization (Sec. 4.5) with high fidelity from a computer-aided design file, the ground truth dimensions of the structures were available for comparison with the microendoscope images. Microendoscope 2 was used for these imaging tests, mounted on a micromanipulator, and aligned over top of the samples for imaging. The printed samples consisted of closely arrayed small spheres, each measuring <10  μm in diameter, forming a plane that overlapped with the oblique light sheet generated by the probe [Fig. S6(a) in the Supplementary Material]. To assess the reconstruction accuracy, we investigated the volume corresponding to 300  μm of sheet propagation distance along the bundle facet (x) and 0 to 150  μm of axial distance from the fiber bundle facet (z). The choice of this range was due to the diminished light sheet intensity outside this region (due to the sheet divergence), which affected the image quality. The image reconstruction was limited by the defocused fluorescence collection of the fiber bundle as z increased and the performance of the reconstruction algorithm. Here, we estimated the lateral resolution to be <10  μm, and the axial resolution was ≈10 to 20  μm for z<70  μm. The averaged reconstructed axial positions are presented in Fig. S6(d) in the Supplementary Material, revealing an additional ≈10 to 50  μm error on the axial positions for z>70  μm.

### 2.3 Light-Sheet Light-Field Fluorescence Imaging

**Fig. 2:**
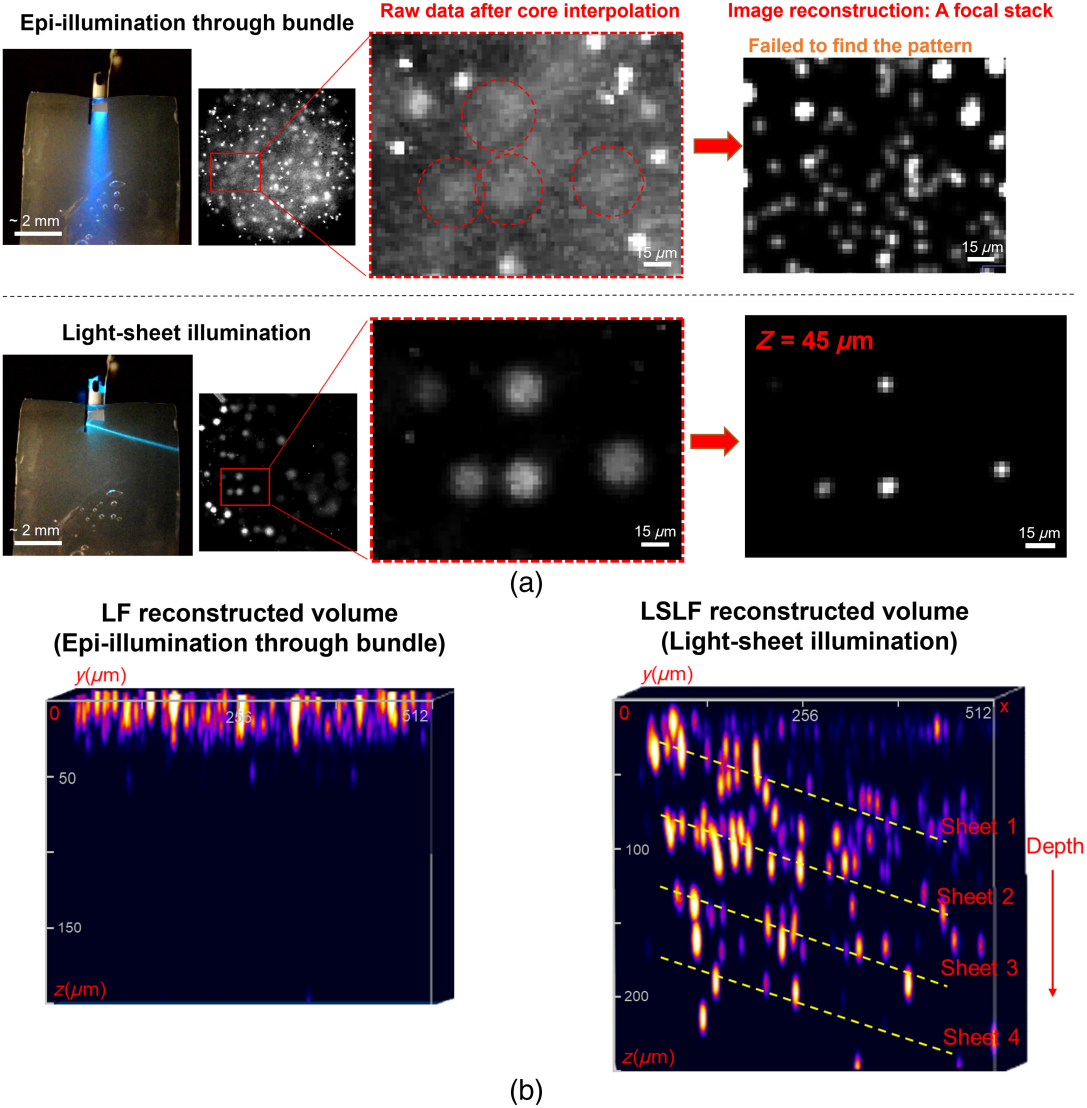
Light-sheet light-field microendoscope imaging of fluorescent beads suspended in agarose. (a) Photographs showing the microendoscope inserted into a block of agarose (left), raw fiber bundle facet images after interpolation between cores (middle), and regions of interest (ROIs) (right) comparing the same cluster of beads using epi- versus light-sheet illumination. The scale bars are 15  μm. (b) Reconstructed volume of 512  μm×512  μm×200  μm (xyz) for epi-illumination (left). Superimposed reconstructed volumes of 512  μm×512  μm×250  μm for sequential illumination from sheets 1 to 4 (right). The dashed yellow lines delineate the estimated trajectories (center lines) of the light sheets based on the beads identified within the reconstructed volumes of each sheet. The z-axis (depth) has been stretched for improved visibility of the beads. (a) and (b) Brightness- and contrast- adjusted to enhance visibility. Microendoscope 1 was used for these imaging tests. The photographs and raw images in panel (a) and the volume reconstructions in panel (b) were adapted from our conference abstract; see Ref. 24.

To validate the volumetric imaging capabilities of our LSLF microendoscopes, we performed a series of imaging experiments with suspended fluorescent beads in agarose and fixed mouse brain tissue samples. The imaging results for the fluorescent beads (yellow-green, ≈2  μm diameter) are summarized in Fig. 2. Microendoscope 1 was used here and fully implanted into the agarose with both the neural probe shanks and fiber bundle facet inserted hundreds of microns into the agarose. Because fiber bundles alone are capable of light-field imaging (via epi-illumination through the fiber bundle),^23^ we used epi-illumination light-field imaging with the fiber bundle in the microendoscope as a convenient benchmark for comparing to our LSLF imaging approach. By imaging the same region of the sample using both methods, i.e., by switching between light-sheet probe illumination and fiber bundle epi-illumination, we observed significant differences. Light-sheet illumination reduced the number of beads per raw image via optical sectioning and greatly enhanced illumination uniformity throughout the image volume, whereas epi-illumination exhibited rapid intensity decay with increasing depth from the fiber bundle facet. These differences are evident in the raw fiber bundle facet images after core interpolation shown in Fig. 2(a), where the epi-illumination raw image contains a multitude of beads spread both laterally and in depth throughout the imaging volume. The bright, well-resolved beads were located very close (<20  μm) to the bundle facet, and a strong, low-contrast background was formed from the superposition of many weakly illuminated out-of-focus beads at larger depths. By contrast, light-sheet illumination, with its inherent optical sectioning, eliminated the background and generated a high-contrast raw image (>5× peak-to-background ratio versus <2× for the same beads with epi-illumination), as shown in Fig. 2(a) and Fig. S9 in the Supplementary Material.

The fiber-bundle light-field deconvolution technique (Sec. 4.4) was used to reconstruct volumetric images from the raw fiber bundle facet images. The resultant reconstructions demonstrate that light-sheet illumination enabled low-noise reconstructions at depths of up to ≈200  μm, whereas low-noise reconstructions with epi-illumination were limited to depths less than about 50  μm. The right panel in Fig. 2(b) presents the superimposed reconstructed volumes of four light sheets (sheets 1 to 4), covering an axial distance from the fiber bundle facet (z), of 0 to 250  μm. Additional images displaying the reconstructed focal stacks for sheets 1 to 4 can be found in Fig. S10 in the Supplementary Material. The dashed yellow lines in Fig. 2(b) denote the estimated light sheet trajectories (center lines) from the beads identified within the reconstructed volumes corresponding to each sheet; these estimates generally agreed with the expected sheet positions based on the GC emitter locations and measured light sheet angles. Sheet 4 extended beyond z=200  μm, resulting in only a few reconstructed beads due to the limited optical power level and increasingly defocused fluorescence collection at greater depths. To evaluate the accuracy of the volumetric image reconstruction, we compared the positions of the reconstructed beads to the sheet positions based on the probe chip geometry. The comparison was performed within the reconstructed volume for z values less than 200  μm and sheet propagation of 300  μm along x. The axial reconstruction accuracy is limited by the defocused fluorescence collection of the fiber bundle and the reconstruction algorithm, and we observed ≈10 to 25  μm of error in the axial positions that increased with z. We estimated the lateral resolution to be ≈10 to 15  μm with this specific sample, though a higher concentration of beads (for a less sparse sample) may reduce the reconstruction quality and lateral resolution.

**Fig. 3:**
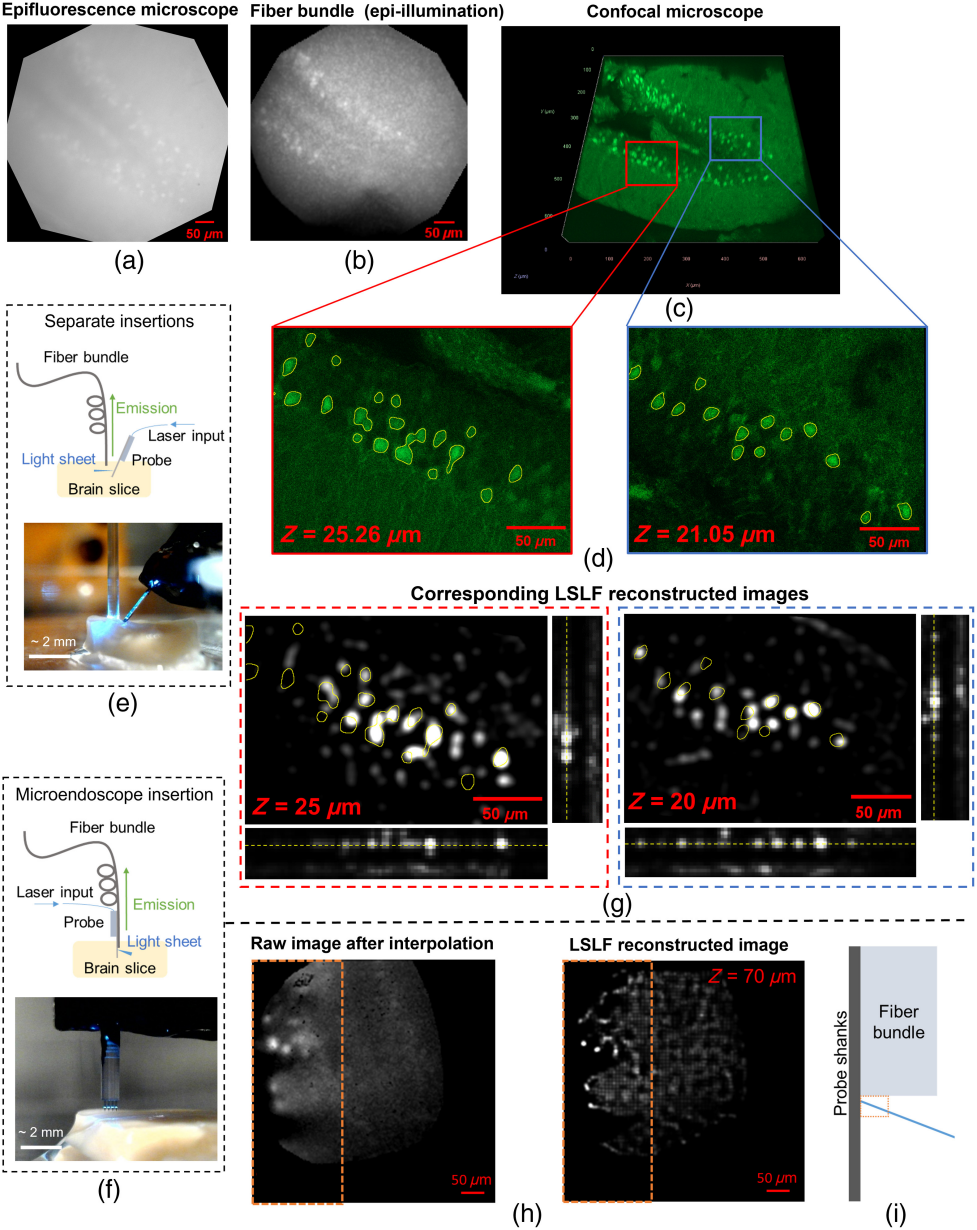
Light-sheet light-field fluorescence imaging of fixed brain tissue from a Thy1-GCaMP6s mouse. (a) Widefield fluorescence microscope image of the target region of the brain slice without the microendoscope. (b) Fiber bundle raw fluorescence image (after core interpolation) of the target region with epi-illumination through the bundle. (c) Volumetric confocal microscope image at the same region with a field of view of 640  μm×640  μm, an axial depth spanning 0 to 50.52  μm, and two ROIs (d) at different depths z (delineated by blue and red rectangles). The yellow overlays in panel (d) indicate neuron soma outlines and were generated via thresholding of the raw images. (e) and (f) Schematics and photographs of the two LSLF imaging geometries investigated: (e) “separate insertions” of the neural probe and fiber bundle (sheets parallel to the bundle facet) and (f) “direct microendoscope insertion” with the probe attached to the bundle (oblique light sheets). Microendoscope 3 was used for the latter imaging tests. Phosphate-buffered saline (PBS) was applied to the surfaces of the brain slices during imaging. (g) LSLF reconstructed images with the separate insertions approach at z=20 and 25  μm. The yellow neuron outlines from (d) (confocal imaging) have been manually aligned and overlaid onto (g). The bars at the bottom and right of (g) indicate maximum intensity projections (MIPs) along both axes (xz- and yz-plane MIPs, z spans 0 to 75  μm). Panels (d) and (g) have been scaled in size and brightness for visibility. Raw images corresponding to (g) are shown in Fig. S11 in the Supplementary Material. (h) Direct microendoscope insertion raw image after core interpolation and LSLF reconstructed image at z=70  μm. (i) Illustration (not to scale) showing a small region close to the shanks and corresponding to the dashed rectangles in panel (h); no patterns consistent with neuron soma were observed outside this region for the direct microendoscope insertion.

Next, we evaluated the LSLF microendoscope imaging characteristics in brain tissue. Here, we imaged a ≈2  mm thick hippocampal fixed brain slice from a transgenic mouse expressing the green fluorescent calcium indicator GCaMP6s (Thy1-GCaMP6s mouse).^8^ The results are summarized in Fig. 3. The brain tissue sample was densely labeled with significant neuropil fluorescence, as observed in the widefield microscope image in Fig. 3(a). This resulted in a prohibitively low image contrast for light-field image reconstruction with epi-illumination through the fiber bundle, and the low contrast is evident in the raw fiber bundle facet image (after core interpolation) in Fig. 3(b).

Photonic neural probe enabled LSLF imaging of the brain slice was tested using two geometries: (1) an image fiber bundle and light-sheet neural probe implanted separately with the probe at an oblique angle such that emitted light sheets were parallel to the fiber bundle facet [Fig. 3(e)] and (2) the fully packaged microendoscope (microendoscope 3) [Fig. 3(f)]. In the first configuration, a light-sheet neural probe was packaged without the image fiber bundle attached, and the neural probe properties were similar to those of the probes in the microendoscopes. The first configuration, with the separately inserted fiber bundle and neural probe, was implemented to avoid the large increase in the sheet depth with propagation distance—a non-ideality of our microendoscope prototypes, which was expected to result in a significant reduction in image quality with sheet propagation in scattering media. Additionally, the neural probe here used a “half-sheet design” with 10 addressable sheets, each spanning 2 adjacent shanks. By contrast, microendoscopes 1-3 used a “full-sheet design” with five addressable sheets, each spanning four shanks. The half-sheet design roughly doubled the sheet intensity for a given input optical power (due to the smaller sheet area), and this was implemented to improve upon the intensity limits observed in microendoscopes 1 to 3. More details of the probe designs are available in our prior work in Ref. 21. To generate an approximate ground-truth image, the region of the brain slice targeted for LSLF imaging was also imaged with a confocal microscope (LSM 900 with Airyscan 2, Zeiss); see Fig. 3(c). As the neural probe and fiber bundle could not be implanted during confocal imaging, tissue displacement from the implantation resulted in shifts in neuronal positions between the LSLF and confocal microscope images, leading to an approximate rather than precise ground-truth image. The time delay between LSLF and confocal microscope images may have also led to some dehydration and tissue shrinkage between the two image sets, further reducing the accuracy of the ground-truth image.

LSLF image reconstructions with the separately inserted fiber bundle and neural probe approach are shown in Fig. 3(g). Neuron outlines generated by thresholding the confocal microscope images were overlaid onto the LSLF images to directly compare the LSLF reconstructions to the approximate ground truth; see Fig. 3(d). We observed approximately 66% and 60% of the neuron outlines matched the LSLF reconstruction for the left and right ROIs shown in Fig. 3(g), respectively. We hypothesize that the discrepancies between the LSLF and confocal images may be due in part to the tissue displacement and shrinkage considerations mentioned above. To simplify this imaging test, the neural probe shanks were inserted into the brain tissue, whereas the fiber bundle facet was only inserted to a shallow, <30  μm depth, with the possibility that the fiber bundle was pressed up against the tissue surface and compressing it rather than being implanted. One of the shanks of the neural probe used here broke during packaging; the corresponding disconnected waveguides at the base of the probe were blocked by the epoxy encapsulation of the probe base to prevent stray light from reaching the brain tissue sample. Because this neural probe used a half-sheet design (with each sheet spanning 2 shanks), only half of the 10 sheets were affected. The sheet used in Fig. 3(g) (left) was not affected, and the sample was illuminated by GC emitters on two shanks. However, the sheet used in Fig. 3(g) (right) was affected, and the sample was illuminated by 1 shank—reducing the sheet area.

For the second LSLF imaging geometry, a microendoscope prototype was implanted into the brain slice with both the neural probe shanks and fiber bundle facet inserted. The reconstructed neurons were primarily located near the shanks, as shown in Fig. 3(h), within a distance of up to 150  μm along the x-axis [Fig. 3(i)]. Beyond this propagation distance, both the raw and LSLF reconstructed images dramatically reduced in quality, a consequence of the increasing sheet depth with propagation distance for this oblique sheet geometry. In both LSLF imaging configurations, these proof-of-concept brain tissue imaging experiments were limited to an imaging depth of about 70  μm, and this is discussed further in the next section.

## 3 Discussion and Conclusion

In this study, we proposed and demonstrated a novel microendoscopic imaging approach using a photonic neural probe for light-sheet illumination and an image fiber bundle for light-field imaging, resulting in a small form factor, implantable, volumetric fluorescence imaging tool. Our imaging approach combines the benefits of fiber bundle light field imaging, i.e., volumetric imaging without a tunable focus element, with the image contrast enhancement of light-sheet illumination. Our proof-of-concept imaging experiments demonstrated a significant enhancement of the reconstructed volumetric images using light-sheet illumination from our neural probes compared with epi-illumination through the fiber bundle. Specifically, the enhanced contrast of the raw fiber bundle facet images enabled larger imaging depths for fluorescent beads suspended in agarose. Also, in a fixed brain tissue sample from a Thy1-GCaMP6s mouse, we observed that light-sheet illumination enabled image reconstruction, whereas epi-illumination yielded no reasonable reconstructed images. Through these initial demonstrations, multiple design and experimental limitations were identified, and in this section, these limitations and potential solutions are outlined. In addition, future extensions of our microendoscopic imaging approach are proposed.

From the outset, a primary limiting factor in the image reconstruction for our LSLF microendoscope is the increasingly defocused fluorescence collection with axial distance—an effect that is magnified by the strong optical scattering of brain tissue. The current prototype microendoscope design further exacerbates this by emitting light sheets at an oblique angle relative to the fiber bundle facet (≈20  deg relative to the normal of the shanks), resulting in an additional axial distance from the fiber bundle facet of ≈110  μm accumulated as the sheet propagates for 300  μm. Thus, the path length of brain tissue that fluorescence must pass through before being collected by the bundle is dependent upon the source location within the sheet, and the excess path length exceeds the optical attenuation length in brain tissue (≈60  μm for green light^39^) for a significant fraction of the sheet. This limitation was particularly evident in the brain tissue imaging experiment with the oblique light sheets in Figs. 3(f) and 3(h), wherein only regions in close proximity to the probe shanks were properly reconstructed. With a simple modification to the neural probe GC emitter design, we expect this effect to be mitigated. The GC, and hence the light sheet, emission angle scales with the grating period, and thus, future iterations of the probe design with smaller GC periods are expected to have light sheet emission angles <5  deg.^40^ The nominal period and duty cycle of the GCs in the prototype neural probe are 400 nm and 50%, respectively, and we have recently demonstrated significantly smaller waveguide feature sizes, albeit in other types of SiN integrated photonic devices.^41^ Aligning the sheets closely to the fiber bundle facet is critical to the image quality as evidenced by the drastically improved image quality of the separately inserted probe and fiber bundle approach in Figs. 3(e) and 3(g) compared with the inline microendoscope, and in sum, decreasing the GC period is expected to increase the image quality while enabling an inline microendoscope form factor.

We hypothesize that the image depths in our brain tissue imaging experiments (about 70μm) were limited in part by the optical power of the light sheets. Unlike typical light-sheet fluorescence microscopes that operate with milliwatts of optical power,^17^^–^^19^^,^^42^ the light sheets emitted from the prototype packaged neural probes in this work (with over 20 dB of optical loss) are over 100× weaker. The fiber-to-chip coupling efficiency was limited to ≈14% (with optimal alignment) due to the use of simple tapered edge couplers with a single SiN waveguide layer.^21^ Recently, we demonstrated low-loss broadband SiN bi-layer edge couplers for visible light,^43^^,^^44^ which have been shown to reduce the coupling loss by 3 to 5 dB compared with the single layer edge couplers in this work at λ=488  nm. We plan to integrate bi-layer edge couplers into our future light-sheet neural probe designs for increased optical transmission. In addition, optimized packaging solutions (e.g., using optical epoxies with less shrinkage) are expected to reduce the transmission variation among the light sheets.

The lateral field of view (FOV) of the LSLF imaging of brain tissue in Fig. 3 was also limited by the optical power of the light sheets, which determined the propagation distance within which the sheet intensity was sufficient for imaging. Precise characterization of this FOV in brain tissue was complicated by the sparsity of labeled neurons and strong neuropil background fluorescence in the brain slices in this work. The cluster of neurons imaged in Fig. 3(g), with a separately inserted neural probe and fiber bundle, was about 150  μm×90  μm, and in the full LSLF reconstructed images (Fig. S11 in the Supplementary Material), fluorescent sources consistent with neurons were reconstructed across an area as large as about 180  μm×190  μm. Here, the FOV was limited by the half-sheet neural probe design used in this experiment. In the raw image in Fig. 3(h) (left), with direct insertion of the microendoscope (using a full-sheet neural probe design), the illuminated area within the dashed box, which encompassed the brightest portion of the light sheet, was about $0.06\;{\mathrm{mm}}^{2}$—roughly 51% of the lateral FOV that we reported in our first demonstration of light sheet neural probes with fluorescence collection via a widefield microscope (240  μm×490  μm).^21^ However, only a small number of neurons were visible close to the shanks due to the oblique sheet geometry discussed earlier, and thus, this area is only an estimate of the illuminated area, not an estimate of FOV over which neurons were reconstructed. In principle, the lateral FOV of fiber bundle light-field microendoscopes can approach the diameter of the bundle^23^ (a 650  μm diameter for the fiber bundles in this work). For our LSLF microendoscope, reaching this limit would require the following improvements: (1) the above-mentioned modifications to the GC design to reduce the light sheet emission angles (for approximately sheet-normal imaging), (2) the previously discussed improvements to the neural probe design and packaging for higher light sheet optical power, and (3) either a reduced fiber bundle diameter that sheets from a single neural probe can traverse or use of multiple neural probe chips in the microendoscope for semi-uniform illumination of a larger area. With these improvements, the potential exists for LSLF microendoscopes to achieve a near-unity ratio of lateral FOV to cross-section area and surpass the ratios achievable with miniature GRIN-lens based microscopes, which have reported FOVs of 500  μm×500  μm to 836  μm×627  μm for GRIN lens diameters of 1 to 2 mm.^5^^,^^26^^,^^37^^,^^38^

The lifetime of our LSLF microendoscopes was determined by the packaging and, specifically, the attachment and alignment of the multicore fiber to the neural probe chip. This alignment was observed to drift with time, reducing the output optical power of the probe. The output power drift was observed to be less than 20% to 30% over 24 h. The lifespan of the packaging of microendoscopes 1 to 3—defined by the point at which the optical power was too weak for fluorescence imaging of the samples in this work—ranged from several days to 2 months. Three microendoscopes were used in this work due to this lifetime limitation. Investigations aimed at enhancing the stability of the packaging are ongoing.

Additional enhancements to the neural probe design to increase the number of light sheets and reduce the sheet thickness are expected to improve the microendoscope performance. The current prototype neural probes have five sheets and were designed as a proof of concept. Full coverage of a 200 to 300  μm depth imaging volume (without gaps) would require a larger number of light sheets. Adding more input edge couplers, more rows of GCs for generating light sheets, and a more complex routing network photonic circuit would allow for a larger number of light sheets. Up to 16 sheets are possible with the multicore fibers that we have demonstrated (which have 10 or 16 cores^34^), and a multicore fiber with a higher core count would support additional sheets. Reducing the light sheet thickness is a complementary design consideration. The thickness of the light sheets in this work was <17  μm over a 300  μm propagation distance in air, which is larger than the ≈5  μm FWHM sheet thicknesses achieved by conventional light-sheet microscopes at λ=488  nm.^17^ In conventional LSFM, thinner sheets result in less background fluorescence collected from out-of-focus planes, and in LSLF imaging, this corresponds to higher sparsity of the raw images and, consequently, improved reconstruction quality. To decrease the thickness of the sheets, a possible approach is to incorporate non-uniform/apodized GC designs, in essence, weakly focusing the emitted light sheets.^45^

Sample labeling sparsity is often a central consideration for computational imaging techniques, and here, sparse samples are expected to improve reconstruction quality and accuracy. The recent emergence of soma-targeted genetically encoded calcium and voltage indicators^11^^,^^46^ is of particular importance to the control of labeling sparsity in brain tissue. Restricting the fluorescent labeling to neuronal soma is a direct approach to increasing the sparsity of the fluorescence images while preserving the critical neuronal activity information and, hence, is an excellent candidate labeling strategy for future LSLF microendoscope functional imaging experiments.

Enhancements to the light-field reconstruction algorithm specific to the LSLF microendoscope may also be possible. By characterization of the microendoscope after packaging, the positions and shapes of the light sheets can be measured, as in Figs. S4 and S5 in the Supplementary Material. With this information, the reconstruction volumes may be precisely tailored to only regions illuminated by light sheets—reducing the computation time. In this work, for LSLF imaging, rectangular reconstruction volumes were used with some tailoring of the volumes based on estimates of the sheet positions and angles. However, further volume reductions are possible, for example, using staircase-shaped reconstruction volumes that closely follow the sheets. In addition, precise characterization of the light sheets may enable significant reductions in the depth error of the reconstructions. In this work, depth information in the reconstructed images was based solely on the light-field reconstruction algorithm, resulting in increasing error in the reconstructed axial positions of fluorescent sources with depth. In future work, the positions of the sheets may be used as an additional source of information to restrict the possible axial positions of fluorescent sources excited by each sheet.

Compared with light-sheet generation with multimode fiber microendoscopes,^47^ our LSLF microendoscope enables light sheets to be generated oblique or orthogonal to the collecting fiber facet, which is advantageous for imaging layered structures and limiting overlapping sources in light-field microscopy. Also, the neural probes provide possibilities for additional microendoscope functionalities, such as electrophysiological recordings and chemical injections via the integration of microelectrodes and microfluidic channels, which we have demonstrated separately in probes for optogenetic stimulation and uncaging in Refs. 36 and 48. However, light-sheet generation with multimode fibers comes with the advantages of relative simplicity in packaging and flexibility in beam profiles (addressable by an external spatial light modulator proximal to the multimode fiber).

In conclusion, the photonic neural probe enabled microendoscope demonstrated herein extends the utility of image fiber bundle light-field imaging via the introduction of patterned illumination in a highly integrated form factor. With optimizations to enhance the illumination geometry and emitted optical power, larger imaging volumes may be possible. In addition, this work paves the way for a variety of new fiber-based computational imaging approaches enhanced by patterned illumination from photonic neural probes, for instance, incorporating a multimode fiber in the microendoscope (in place of the image fiber bundle) while adapting recently demonstrated multimode fiber based photometry and computational imaging approaches.^49^^–^^53^ Though our imaging demonstrations were limited to fluorescent beads suspended in agarose and fixed brain tissue, the microendoscope operating principles, image reconstruction results, observed limitations, and assembly technique detailed here provide a foundation for future explorations of this technology for deep-brain fluorescence imaging *in vivo*.

## 4 Appendix: Methods

### 4.1 Imaging Apparatus

The apparatus for imaging the proximal facet of the image fiber bundle is shown in Fig. 1(a) and consists of an epifluorescence microscope (Cerna Microscope, Thorlabs) with an infinity-corrected long working distance objective (Plan Apochromat, 20X, NA=0.42, working distance 20 mm, Mitutoyo Corporation), a GFP filter cube (emission filter, MF525-39, Thorlabs), and a back-illuminated sCMOS camera (Prime BSI, Teledyne). We used 3′ and 4′ long Fujikura FIGH-30-650S image fiber bundles throughout the experiments. Each fiber bundle had an SMA connector on one end, and the other end was polished before the packaging process. The image fiber bundles had a diameter of 650±30  μm (750±30  μm prior to removal of the coating), 30,000 cores, an average core diameter of about 2  μm, and average core-to-core spacing of about 3.2  μm.^23^ The fiber bundle cores supported ≈11 modes at λ=515  nm.^23^ The proximal end of the fiber bundle was mounted on a three-axis translation stage fixed to the epifluorescence microscope objective, ensuring stability in the position of the facet relative to the objective and, hence, the image focus. For imaging experiments, the distal end of the microendoscope was mounted on a four-axis micromanipulator (uMP-4, Sensapex) for insertion into samples. For experiments using epi-illumination through the fiber bundle, blue epi-illumination from the epifluorescence microscope (used to image the proximal fiber bundle facet) was coupled into the fiber bundle; blue light was input to the microscope from a light-emitting diode (LED) source (pE-4000, CoolLED).

### 4.2 Light-Sheet Light-Field Microendoscope Packaging

We followed a three-step process to package each microendoscope: (1) fiber bundle attachment to the light-sheet neural probe, (2) laser coupling, and (3) multicore fiber attachment to the neural probe. Detailed figures showing the packaging process are presented in Note 1 in the Supplementary Material. First, the light-sheet probe was glued on a metal probe holder/carrier using 5 min epoxy (1365868, Loctite), and the probe holder was mounted with the probe surface upright under a microscope. An image fiber bundle was positioned above and in close proximity to the probe (parallel to the shanks) [Fig. S1(b) in the Supplementary Material] with the fiber bundle facet close to the most proximal row of GCs (corresponding to sheet 1); see Fig. S1(c) in the Supplementary Material. The fiber bundle was fixed to the neural probe by applying UV-curable epoxy (OP-67-LS, DYMAX) and curing with a UV LED system (CS2010, 365 nm, Thorlabs). Next, a custom visible-light multicore fiber^34^ (10 cores, Corning) with cleaved facets was connected and aligned to a computer-controlled optical scanning system^35^^,^^36^ [Fig. S2(a) in the Supplementary Material]. The scanning system used a microelectromechanical system (MEMS) mirror to deflect a collimated beam from a 488-nm laser (OBIS 488 nm LX 100 mW, Coherent Inc.) at an addressable angle and focus it to a spot with an addressable position along the fiber facet, enabling computer-controlled selection of different cores of the multicore fiber; see Fig. S2(b) in the Supplementary Material. The average transmission of the scanning system was ≈60% at λ=488  nm. Finally, the multicore fiber was aligned to the light-sheet probe base with each core coupled to an on-chip fiber-to-chip edge coupler; see Fig. S2(c) in the Supplementary Material. UV-curable optical epoxies (OP-4-20632 and OP-67-LS, DYMAX) were used to attach the multicore fiber to the probe base and encapsulate the connection (Fig. S3 in the Supplementary Material). The body of the fully packaged microendoscope was encapsulated with optically opaque epoxy (EPO-TEK 320, Epoxy Technology Inc.) to block stray light incident on the probe chip facet that was not coupled into the on-chip waveguides. The light-sheet neural probe used for LSLF brain tissue imaging via a separately inserted probe and fiber bundle, shown in Fig. 3(e), was packaged similarly but without the fiber bundle attachment step. The 488-nm laser and scanning system were used throughout the imaging experiments for input light and sheet addressing for the neural probes.

### 4.3 Light Sheet Characterization

The light sheet profiles from the packaged neural probe in microendoscope 1 were characterized by the methods used in our previous work.^21^ First, the probe shanks were immersed in a fluorescein solution, as shown in Fig. S4 in the Supplementary Material. A microscope with a fluorescence emission filter was positioned at a flat sidewall of the fluorescein chamber, and via imaging of the resultant fluorescence from the light-sheet excitation, side profiles of the light sheets were acquired. The additional upward-pointing beams observed in the beam profiles were due to second-order diffraction from the GC emitters.^54^ The second-order diffraction was only visible at high laser powers and had optical power several times lower than the light sheets (first order diffraction). Next, the light sheets were characterized in air by positioning a coverslip with a fluorescent thin film above and in close proximity to the GC emitters; see Fig. S5 in the Supplementary Material. The incident light sheet excited the fluorescent thin film, and the resultant fluorescence was imaged with a widefield microscope above the coverslip. The relative height of the thin film above the neural probe was controlled with a translation stage, and the FWHM sheet thicknesses were calculated at multiple thin film positions and, hence, light sheet propagation distances.

### 4.4 Light Field Image Reconstruction

To reconstruct volumetric images from raw proximal fiber bundle facet images, we applied the fiber-bundle light-field imaging algorithm proposed and demonstrated in Ref. 23. The flowchart of the volumetric image reconstruction process is detailed in Fig. S7 in the Supplementary Material. To initially test the algorithm and our software implementation, we imaged yellow-green fluorescent microbeads (FluoSpheres Polystyrene, 10  μm, Thermo Fisher Scientific Inc.) dried onto the surface of a glass slide using a fiber bundle without a neural probe attached. Epi-illumination through the bundle was used to illuminate the sample, and here, blue LED light was coupled into the fiber bundle from the epifluorescence microscope used to image the fiber bundle proximal facet. The fluorescent sample was placed on a piezoelectric stage (LSP710E/M, Thorlabs) to control the distance between the glass slide and fiber bundle facet. Raw and reconstructed images of the fluorescent beads positioned at distances of z=10, 30, and 50  μm from the fiber bundle facet are shown in Fig. S8 in the Supplementary Material. The standard shift-and-add light field refocusing technique^55^ was used as a step in the volume reconstructions; see Figs. S8(d)–S8(f) in the Supplementary Material. However, this step alone did not resolve the axial locations [Figs. S8(g)–S8(i) in the Supplementary Material]. To resolve the micro-beads axially, we subsequently applied deconvolution of the refocused axial stacks,^23^^,^^56^ as shown in Figs. S8(j)–S8(o) in the Supplementary Material; some features that were merged in the raw images (Fig. S8 in the Supplementary Material, delineated by red rectangles) were resolvable in the deconvolved focal planes. The reconstructed volumetric image had <10  μm of lateral resolution with ±6  μm of depth accuracy for axial distance z of up to 60  μm.

For light-sheet neural probe illumination, a volumetric reconstruction was performed for each sheet. The reconstruction volumes were rectangular with sizes chosen to contain their respective light sheets (based on estimates of the light sheet positions and angles). Additional volume was included to account for the error in the sheet position and angle estimates. All image reconstructions were performed in Matlab R2020b (Mathworks). Figure 2(b), the neuron outlines in Figs. 3(d) and 3(g), and the intensity line profile in Fig. S6(c) in the Supplementary Material were generated in Fiji.^57^

### 4.5 3D-Printed Samples for Microendoscope Resolution Estimates

Samples for microendoscope resolution estimates were fabricated using a Nanoscribe Photonic Professional GT2 two-photon polymerization 3D printer. The samples were printed in IP-S resin (Nanoscribe GmbH), an inherently autofluorescent resin that enables high-precision 3D printing of microstructures. We printed two different samples [Fig. S6(b) in the Supplementary Material]: a sparse structure with an axial resolution (sphere-to-sphere spacing) of 20  μm and a fine structure with a lateral and axial resolution of 10  μm for evaluating the image reconstruction. The printing parameters and development time were optimized for the high aspect ratios and small features of these structures.

### 4.6 Fluorescent Beads in Agarose

Agarose gel blocks were prepared by mixing agarose powder (UltraPure Agarose, Invitrogen) with deionized water to form a 1% agarose solution. Yellow-green fluorescent microbeads (FluoSpheres carboxylate, 2  μm, Thermo Fisher Scientific Inc.) were added to the agarose mixture, which was poured into a rectangular mould and solidified in a refrigerator.

### 4.7 Fixed Brain Tissue Preparation

A fixed whole brain from a Thy1-GCaMP6s (GP4.12Dkim/J) transgenic mouse^8^ (The Jackson Laboratory, stock number 025776) was purchased from BrainBits by Transnetyx Inc. (New York, United States). The brain was fixed in 1% to 1.5% paraformaldehyde (PFA) for about 8 h overnight and stored in phosphate-buffered saline (PBS). Before experiments, 2-mm thick brain slices were prepared with a brain matrix (Alto Brain Matrix stainless steel 1 mm mouse coronal 45 to 75 gm; Harvard Apparatus) and stirrup-shaped blades (Type 102, Carl Roth GmbH + Co. KG). PBS was added to the surfaces of the fixed brain tissue slices during imaging experiments to prevent drying of the tissue.

## Data Availability

The codes generated during this study with example data for a single layer of fluorescent beads are available at GitHub with the following link: https://github.com/Pding2/Light-field-fiber-bundle-imaging.git All data generated during this study and all of the raw data are available from the corresponding authors upon request.
