# Supplementary material for: Photonic neural probe enabled microendoscopes for light-sheet light-field computational fluorescence brain imaging
Source: Neurophotonics. 2024 Feb 6;11(Suppl 1):S11503. doi: 10.1117/1.NPh.11.S1.S11503 (PMC10846542; doi:10.1117/1.NPh.11.S1.S11503)
Supplement: Supplementary file 1 [file NPh_011_S11503_SD001.pdf]

Supplementary material: Photonic neural probe enabled microendoscopes for light-sheet light-field computational fluorescence brain imaging

Peisheng Ding[a,b,*], **Hannes Wahn**[a], **Fu-Der Chen**[a,b,c], **Jianfeng Li**[a,c], **Xin Mu**[a,b,c], **Andrei Stalmashonak**[a], **Xianshu Luo**[d], **Guo-Qiang Lo**[d], **Joyce K. S. Poon**[a,b,c], **Wesley D. Sacher**[a,c,*]
[a]Max Planck Institute of Microstructure Physics, Weinberg 2, Halle, 06120, Germany
[b]Department of Electrical and Computer Engineering, University of Toronto, 10 King's College Road, Toronto, Ontario, M5S 3G4, Canada
[c]Max Planck-University of Toronto Centre for Neural Science and Technology
[d]Advanced Micro Foundry Pte. Ltd., 11 Science Park Road, Singapore Science Park II, 117685, Singapore
[*]Peisheng Ding, peisheng.ding@mpi-halle.mpg.de, Wesley D. Sacher, wesley.sacher@mpi-halle.mpg.de

Note 1. Light-sheet light-field (LSLF) microendoscope packaging: Figs. S1-S3.
Note 2. Microendoscope characterization: Figs. S4-S6.
Note 3. Light-field image reconstruction: Figs. S7-S8
Note 4. LSLF microendoscope fluorescence imaging: Figs. S9-S11.

Note 1. Light-sheet light-field (LSLF) microendoscope packaging

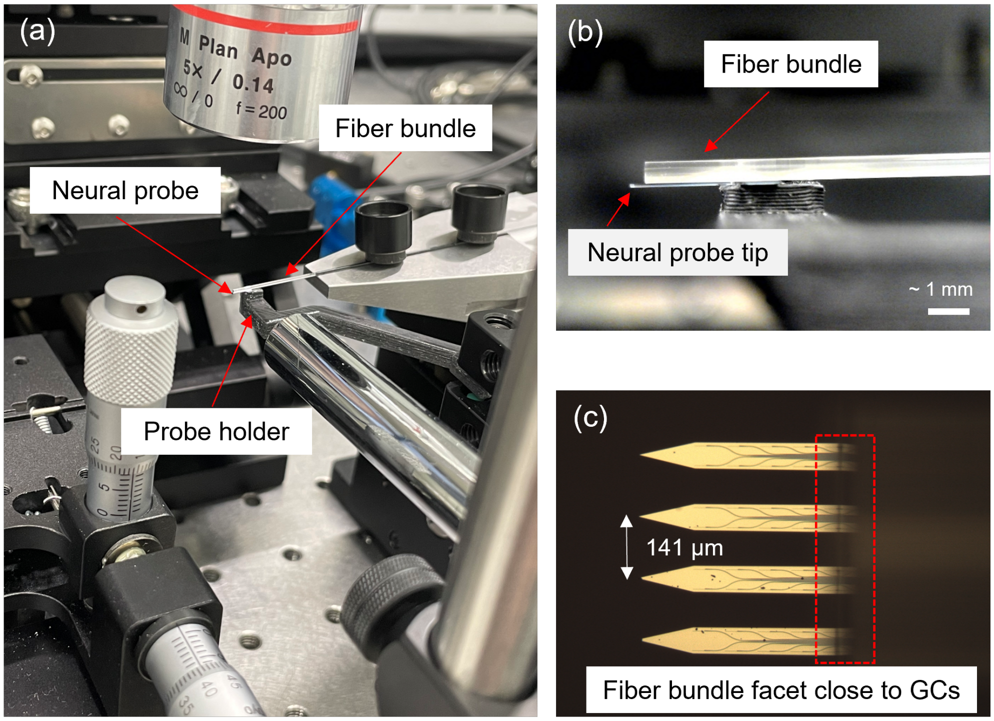

Fig S1 Microendoscope fiber bundle attachment. (a) Photograph showing attachment of an image fiber bundle to a photonic neural probe and the corresponding apparatus. The neural probe was fixed onto a probe holder/carrier using 5 min epoxy and the probe holder was mounted under a microscope. (b) Side-view photograph of the next step in the process where the fiber bundle was aligned over top of the neural probe using a translation stage. (c) Top-down optical micrograph of the microendoscope tip following the next step where the fiber bundle was epoxied to the neural probe. The neural probe tip is visible in (b) where the fiber bundle is placed parallel to and on top of the shanks, while ensuring the bundle facet is in close proximity to the grating coupler (GC) emitters, and specifically, in close proximity to the most proximal row of GCs (Sheet 1). The attachment was encapsulated by UV epoxy.

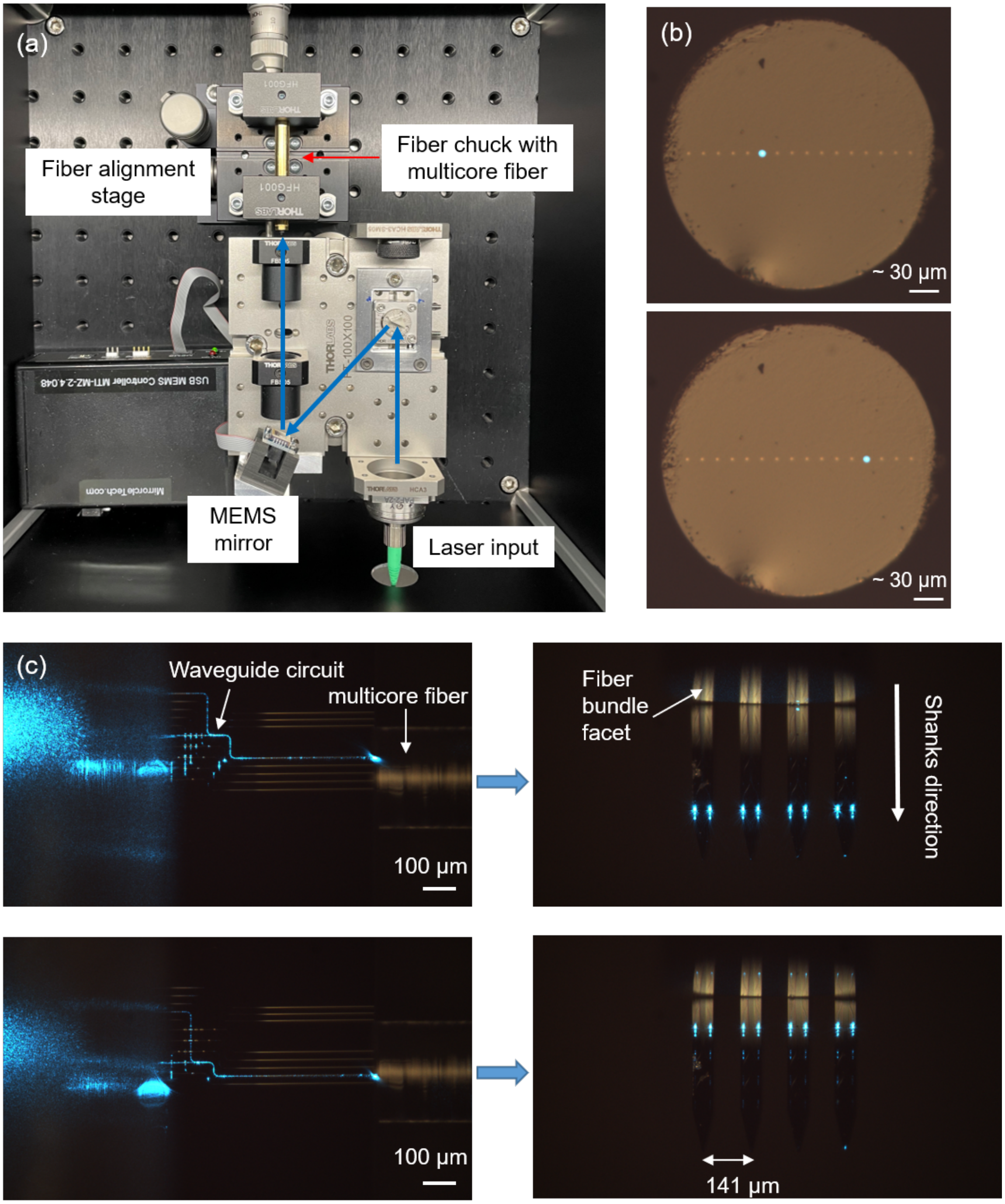

Fig S2 Visible-light multicore fiber alignment. (a) Top-down photograph of the optical scanning system used to address the cores of the multicore fiber. (b) Optical micrographs of the facet of a multicore fiber coupled to the scanning system; light is coupled into different cores of the fiber via actuation of the MEMS mirror (spatial addressing). A multicore fiber with 16 cores is shown here as an example, and 10-core fiber was used in the microendoscopes. (c) Top-down optical micrographs of the neural probe chip facet (left) and shanks (right) with the multicore fiber aligned to the array of fiber-to-chip waveguide edge couplers on the neural probe. When selected by the scanning system, each core of the multicore fiber couples laser light to an edge coupler of the neural probe (left) and the light is routed to a row of GCs on the shanks where it is emitted as a light sheet (right).

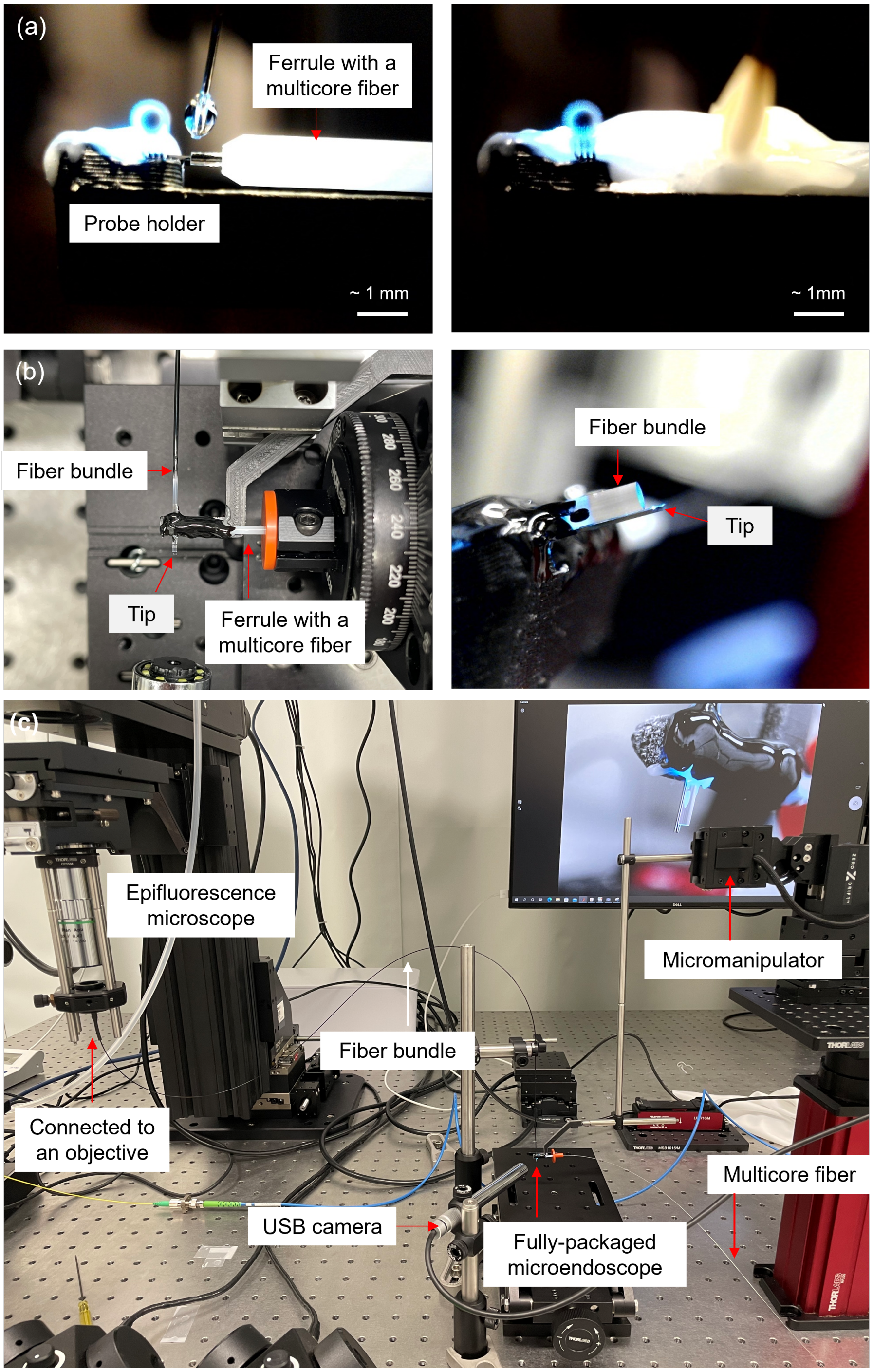

Fig S3 Multicore fiber attachment. (a) Side-view photograph during application of UV epoxy to attach the multicore fiber to the neural probe chip facet. The multicore fiber was fixed in a ferrule for stability of the alignment and strength of the final package. (b) Top- and side-view photographs after completion of the packaging process. (c) Photograph of the microendoscope imaging system. Laser light is coupled into the neural probe through the multicore fiber and the proximal end of the fiber bundle is imaged with an epifluorescence microscope. An image of the fully-packaged microendoscope captured by a USB camera is shown on the background screen. A micromanipulator was used to control the microendoscopic insertion into samples.

Note 2. Microendoscope characterization

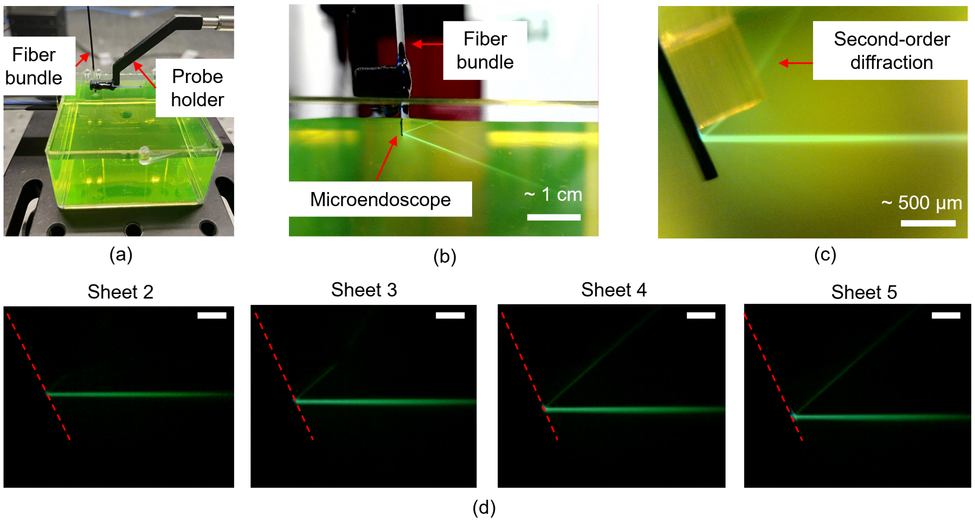

Fig S4 Light sheet characterization in a fluorescein solution. (a) and (b) Photographs showing the distal end of the packaged LSLF microendoscope immersed in a fluorescein solution. (c) Side-view photograph of the resultant fluorescence from a light sheet emitted by the microendoscope into the fluorescein solution. (d) Fluorescence images of the light sheet profiles of the microendoscope. The dashed red lines delineate the top surface of the shanks. Scale bars: 200 µm. The sheet thicknesses in these side profile measurements are overestimated due to the collection of out-of-focus light by the microscope (i.e., the sheet width was larger than the microscope depth of focus). The secondary upward-pointing beams observed in (b), (c), and Sheets 3 to 5 in (d) were due to second-order diffraction from the GC emitters as discussed in Sec. 4.3 of the manuscript. These beams were only visible at high laser powers and their optical powers were approximately 7×, 5×, and 4× lower than the those of the primary light sheets for Sheets 3 to 5, respectively. Microendoscope 1 was used for the light sheet characterization in fluorescein.

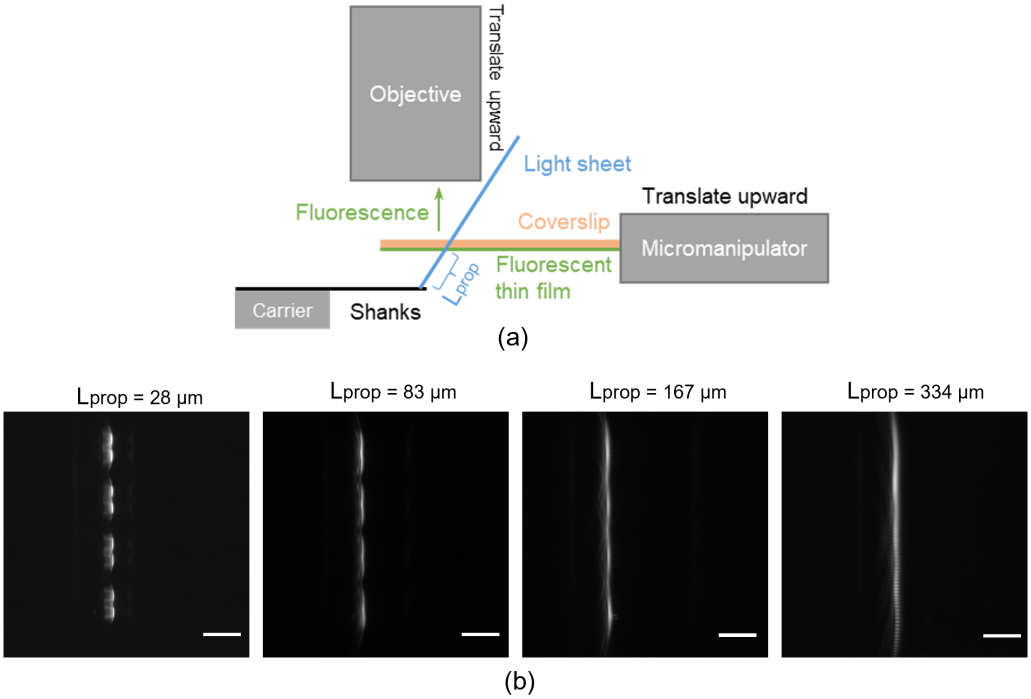

Fig S5 Free-space light-sheet profile measurements. (a) Illustration of the light sheet profile measurement approach (not to scale). A coverslip coated with a fluorescent thin film on one side was positioned above and parallel to the neural probe shanks. A light sheet emitted by the neural probe was incident on the thin film, and the resultant fluorescence, corresponding to a cross-section of the light sheet, was imaged by a widefield microscope above the coverslip. The distance between the fluorescent thin film and the neural probe was varied to image the light sheet cross-section at various propagation distances. (b) Light sheet cross-sections imaged at discrete light sheet propagation distances, L_{prop}. All images have been intensity normalized for visibility. Scale bars: 100 µm. The optical emissions from the row of GCs corresponding to the light sheet merged with increasing propagation distance, synthesizing a semi-uniform sheet with averaged FWHM thickness < 11 µm, < 17 µm, < 20 µm for L_{prop} of 100 µm, 300 µm, and 500 µm, respectively. Microendoscope 1 was used for the light sheet characterization in free space.

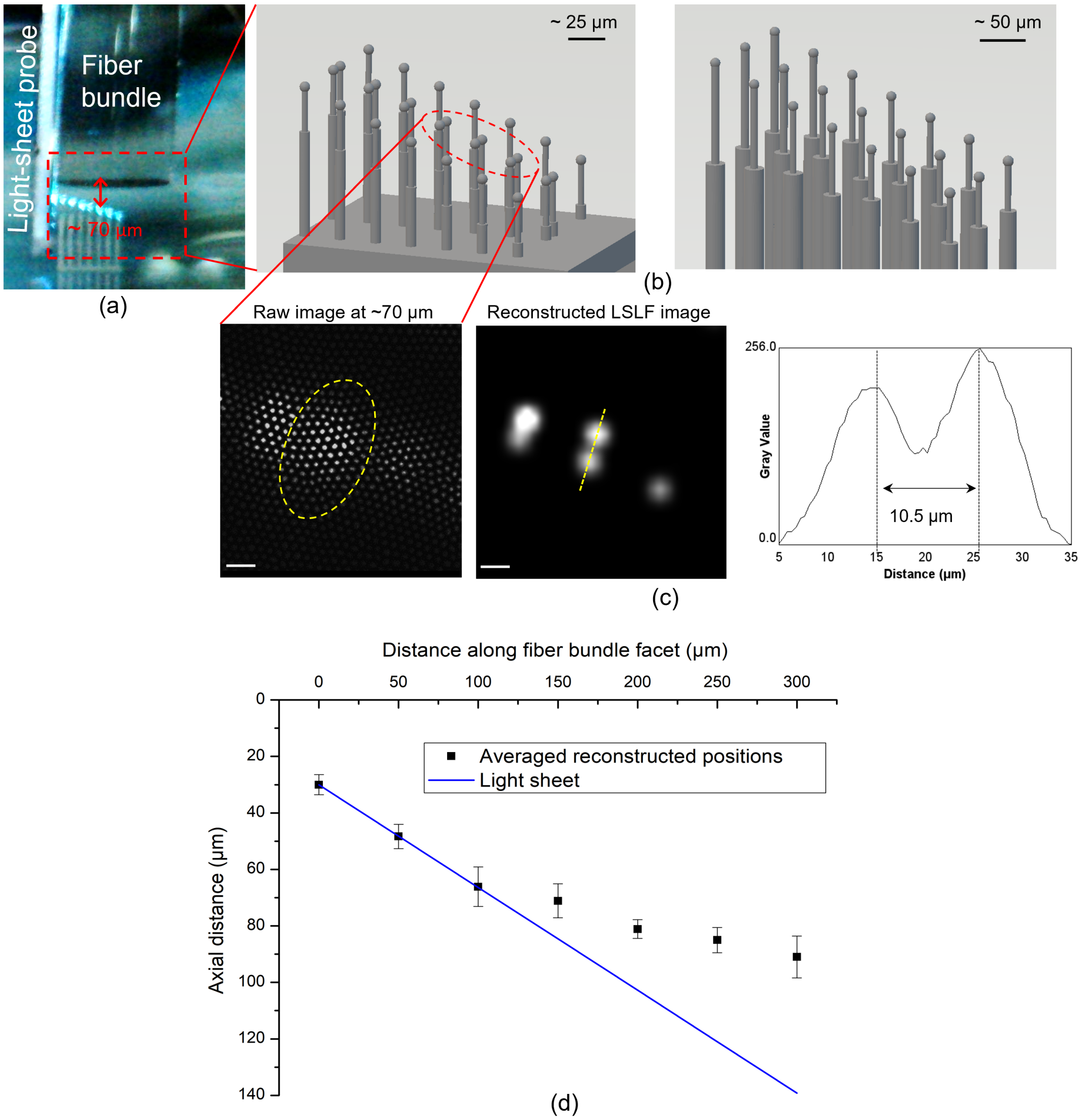

Fig S6 Microendoscope resolution estimates via imaging of 3D-printed microstructures. (a) Side-view photograph of a 3D-printed microstructure being imaged by the LSLF microendoscope (Microendoscope 2). The microstructure consisted of closely-arrayed small spheres, forming a plane that overlapped with the oblique light sheet emitted by the microendoscope. (b) Computer-aided design (CAD) drawings of the two microstructures used in this work: a fine structure (left) containing axial (z) and lateral (x) spacing between spheres of 10 µm; a sparse structure (right) with a z-spacing of 20 µm. The microstructures were fabricated by two photon polymerization 3D printing (Nanoscribe Photonic Professional GT2), and more details of the printing process are described in Sec. 2.2 and 4.5 of the manuscript. (c) Raw microendoscope images collected at the proximal fiber bundle facet (left) of a region of interest (ROI) with 2×3 spheres at an axial distance (z) of ≈70 µm from the distal fiber bundle facet. Reconstructed LSLF image (right) with intensity profile of the marked line, indicating the resolvable 10 µm spacing. Scale bars: 10 µm. (d) Graph showing the z-position of Sheet 1 along the x-direction based on the measured light sheet geometry and the averaged reconstructed axial depths (for the sparse structure), showing ≈ 10-50 µm of error on the axial positions when z is greater than 70 µm.

Note 3. Light-field image reconstruction

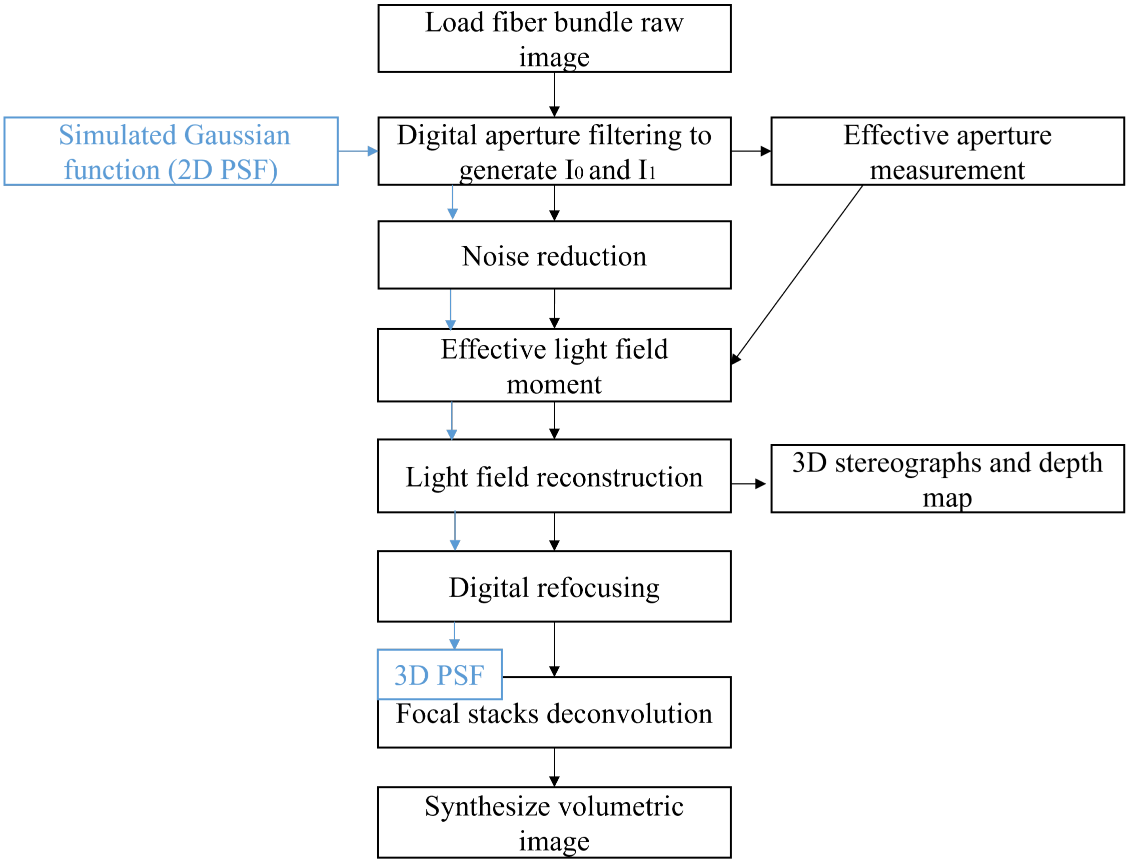

Fig S7 Light-field image reconstruction algorithm. The flowchart summarizes the reconstruction algorithm applied in this work and is adapted from Refs. 23, 30, 31, 55, and 56. The input is a single raw image of the proximal fiber bundle facet of the microendoscope captured by the epifluorescence microscope camera, and the output is the synthesized volumetric image stack. The first step is to load the raw image and perform digital aperture filtering [23] to generate two images, I_0 (small-aperture image) and I_1 (full-aperture image), followed by noise reduction. Next, the effective light field moment is calculated in Fourier space to retrieve the averaged angular distribution (u,v) of the input light, which is then used to reconstruct a light field L(x, y, u, v) [23,30]. The parallax in the light field can be used to create perspective-shifting animations and 3D stereo images, as well as the depth map by using the least-squares equation [31]. The refocused focal stacks are created by the digital refocusing technique [55] and deconvolved with a simulated point spread function (PSF) [23,56].

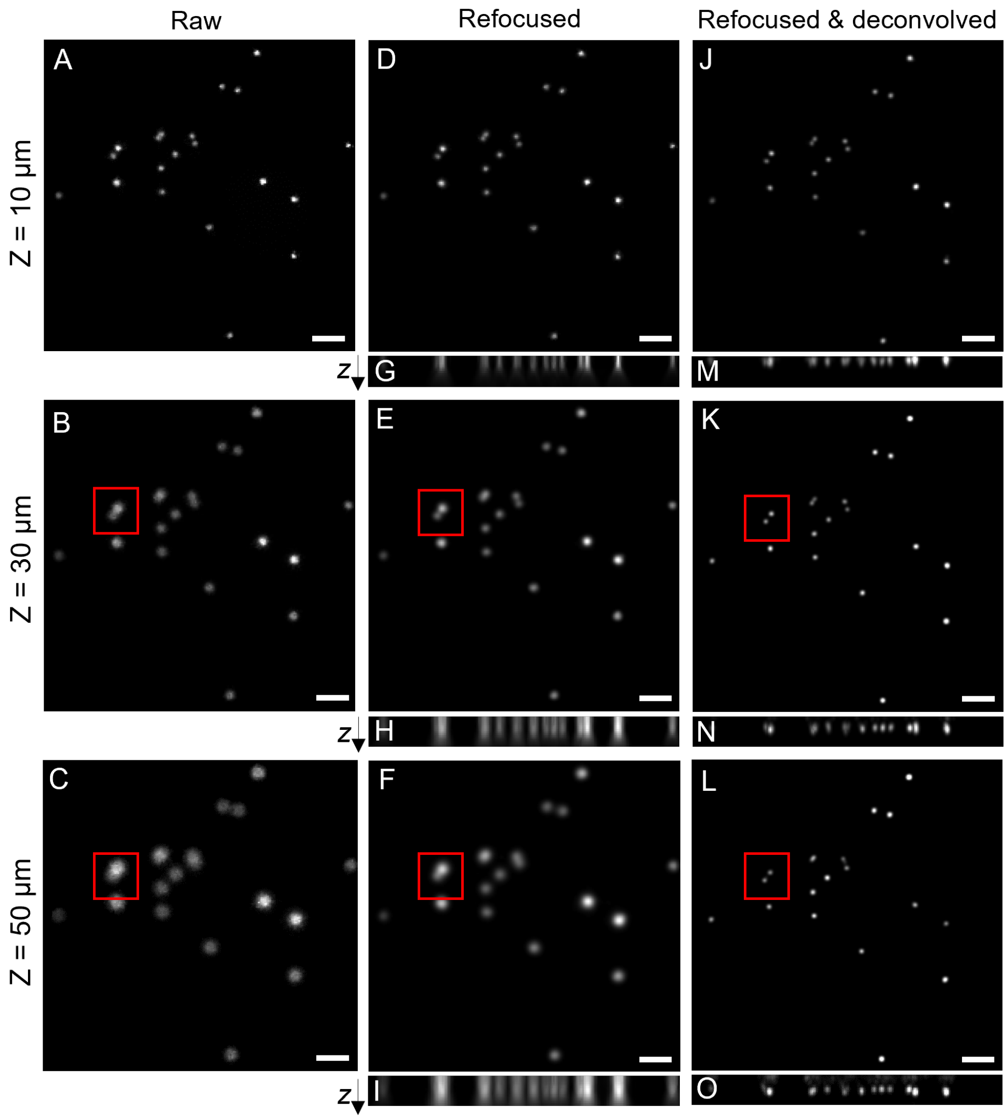

Fig S8 Light-field image reconstruction tests with epi-illumination from a fiber bundle. A fluorescent microbead sample was placed at a distance of z = 10, 30, and 50 µm (ground truth planes) from the fiber bundle facet and epi-illumination was applied through the fiber bundle. (A-C) Raw images after fiber-bundle core interpolation. The defocused fluorescence collection blurs the beads. (D-F) Refocused images after applying the digital refocusing technique. (G-I) xz-plane maximum intensity projections (MIPs) of the refocused image stacks in (D-F), respectively. The arrow indicates the z distance, from 0 to 102.2 µm. (J-L) xy-plane MIPs of deconvolved focal stacks of the same sample. (M-O) xz–plane MIPs of the deconvolved focal stacks in (J-L), respectively. Scale bars: 50 µm. The red rectangles highlight features that were merged in the raw and refocused images, but resolvable in the deconvolved focal planes. All images have been intensity adjusted for visibility. We conducted this experiment for initial testing and validation of the light-field image reconstruction algorithm.

Note 4. LSLF microendoscope fluorescence imaging

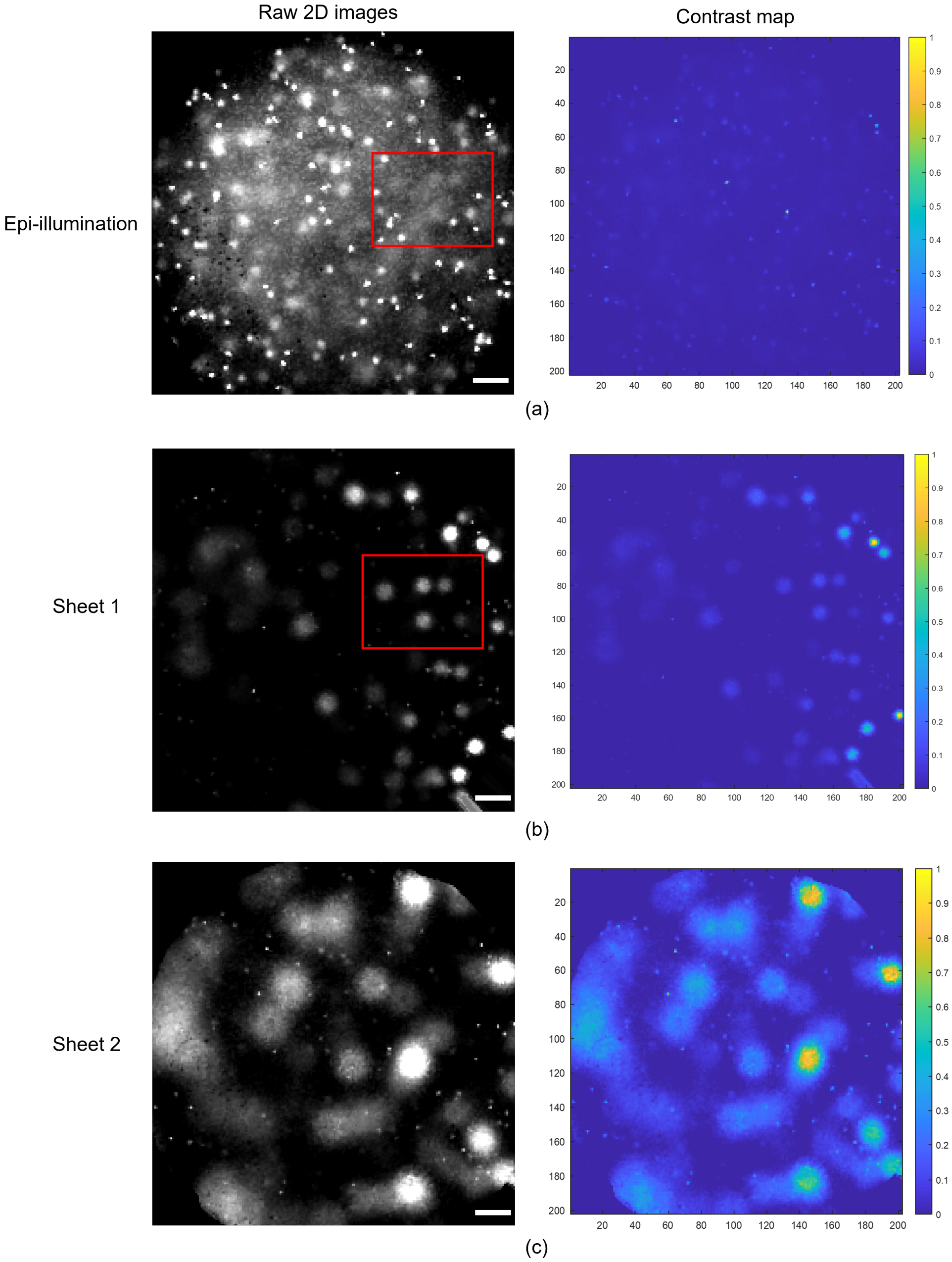

Fig S9 Microendoscope raw images (after fiber-bundle core interpolation) of fluorescent beads suspended in agarose. Epi-illumination through the fiber bundle and light-sheet neural probe illumination are compared. The microendoscope was first inserted into the sample, and epi-illumination and light-sheet images were captured at the same position. The raw fiber bundle facet images and contrast maps for (a) epi-illumination, (b) Sheet 1, and (c) Sheet 2 are shown. Scale bars: 50 μm. The contrast maps span areas of 200 pixels x 200 pixels; the pixel numbers are labeled on the axes and each pixel has a side length of 2.56 μm. The images in the first column have been intensity-scaled for visualization of the background fluorescence, and the contrast maps in the second column have been normalized to the maximum value. The red rectangles delineate ROIs showing similar features in (a) and (b). Microendoscope 1 was used for these imaging tests.

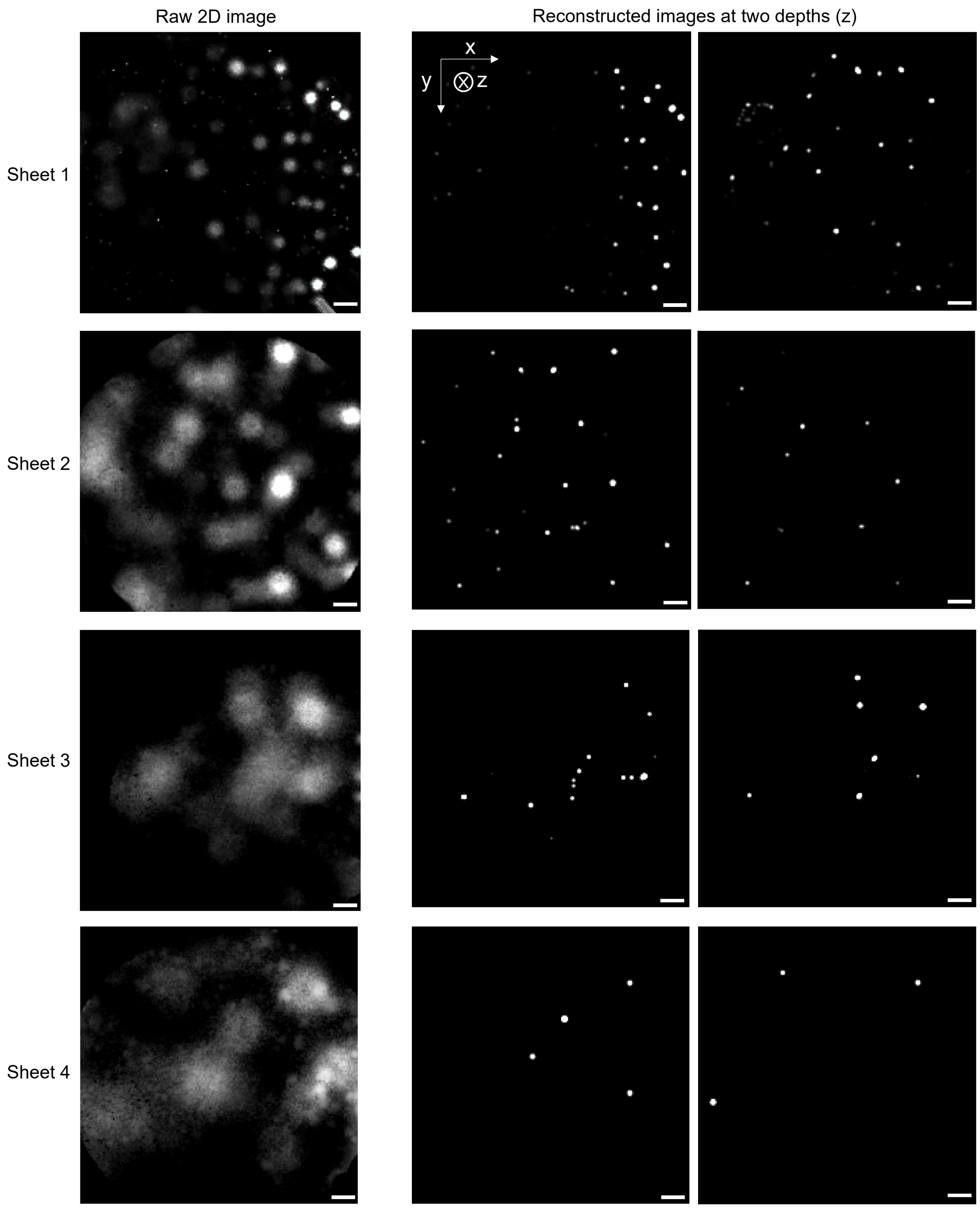

Fig S10 Microendoscope LSLF images of fluorescent beads suspended in agarose. Images corresponding to Sheets 1 to 4 are shown in the first to fourth rows, respectively. The first column shows the raw images after fiber bundle core interpolation; the second and third columns present reconstructed images at two arbitrary depth planes of the focal stack with ≈30 µm axial (z) spacing. The x, y, and z-directions are indicated. All images have been intensity-adjusted for visibility. Scale bars: 50 µm. Microendoscope 1 was used for these imaging tests.

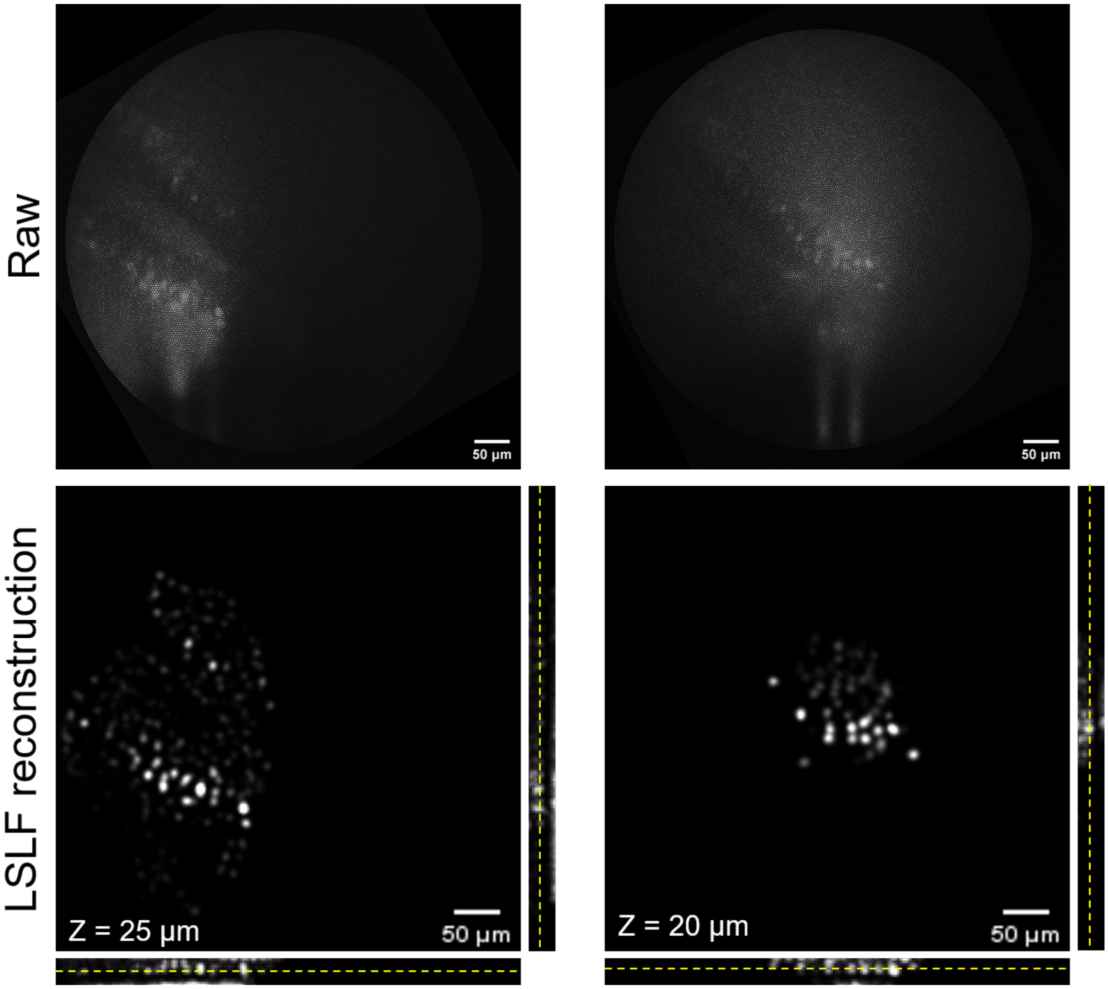

Fig S11 Microendoscope LSLF imaging of fixed brain tissue with separate insertions of the neural probe and fiber bundle (sheets parallel to the bundle facet). Raw and reconstructed images spanning the entire imaged area of the proximal bundle facet are shown. These images correspond to the reconstructions in Fig. 3(g) with the left (right) column corresponding to Fig. 3(g) - left (right). The raw images (first row) are 655 μm × 655 μm. The LSLF reconstructed images (second row) are 512 μm × 512 μm and correspond to depths beyond the bundle facet of z = 20 and 25 μm. The bottom and right bars are maximum intensity projections (MIPs) along both axes (xz- and yz-plane MIPs, z spans 0 to 60 μm). All images were scaled in brightness for visibility. To optimize the reconstructions for the larger area here vs. Fig. 3(g), the point spread function (PSF) used for deconvolution was slightly modified. The reconstructions in the bottom row used a PSF with a depth span of 0 - 60 μm, while Fig. 3(g) used a PSF depth span of 0 - 75 μm. The shanks are located at the bottom of the images with the sheets propagating from the bottom to the top. As noted in Sec. 2.3 of the manuscript, a neural probe with a "half-sheet" design (with each sheet spanning 2 adjacent shanks) was used here. One of the shanks broke during packaging, affecting half of the sheets. The images in the left column were not affected, and the sample was illuminated by a sheet spanning 2 shanks. However, the images in the right column were affected, and the sample was illuminated by a sheet generated by 1 shank.
